# Commentary: An *In Silico* – *In Vitro* Pipeline Identifying an HLA-A*02:01^+^ KRAS G12V^+^ Spliced Epitope Candidate for a Broad Tumor-Immune Response in Cancer Patients

**DOI:** 10.3389/fimmu.2021.523906

**Published:** 2021-07-13

**Authors:** Ilan Beer

**Affiliations:** Adicet Bio Inc., Menlo Park, CA, United States

**Keywords:** peptide splicing, proteasome, KRAS mutation, HLA, mass spectrometry

## Introduction

In their original research (1), the authors were looking for a potential KRAS epitope for use in targeted cancer immunotherapy in the form of an HLA-presented peptide that would have the following three properties: i) cover the KRAS G12V mutation; ii) be a result of peptide digestion and splicing in the proteasome; iii) bind to HLA-A*02:01. The authors claim to have found such a peptide (KLVVGAVGV) by *in vitro* digestion of a mutated KRAS polypeptide by purified proteasomes. However, we suggest that this finding may have been based on misidentification of the peptide. Our analysis and conclusion relied on data deposited by the authors in PRIDE project PXD015580, including mass-spectrometry (MS) raw files and peptide identification results.

The authors initially performed 45 MS analyses of the proteasome-digested KRAS polypeptides. These included 3 biological replicates x 3 technical replicates, where each of the 9 replicates was sampled for MS analysis at 5 time points (0, 1, 2, 3 and 4 hours). Several weeks later, the authors performed two additional MS analyses of the peptides after 20 hours of digestion, and two runs of two synthetic peptides, including the peptide in question.

## Spectra Identified by the Authors as the Spliced Peptide KLVVGAVGV May Have Been Misidentified

**Figure 1:**
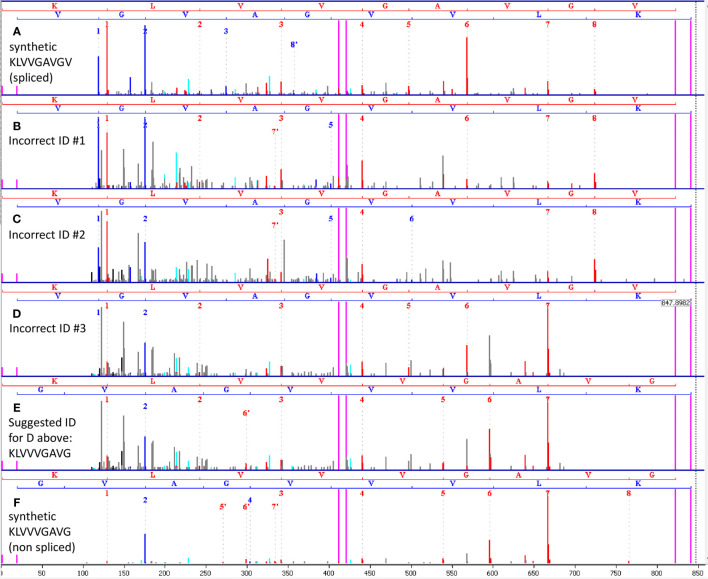
**(A)** Spectrum of synthetic KLVVGAVGV, obtained from the authors’ data. This synthetic peptide represents the proteasome-spliced peptide claimed to have been found by the authors by MS. **(B–D)** Three representatives of the multiple spectra identified by the authors as the spliced peptide KLVVGAVGV in the first 45 runs. Each of the other spectra is similar to one of these representatives. None of the spectra looks like the synthetic spectrum in **(A)**. Hence, we may conclude that the spliced peptide KLVVGAVGV was not present in the 45 runs. **(E)** Our suggested alternative identification of the spectrum in **(D)** as KLVVVGAVG, a non-spliced peptide. **(F)** Spectrum of synthetic KLVVVGAVG confirms the alternative identification in **(E)**. The spectra in **(B, C)** were found in time 0, and hence cannot be proteasome products. In **(A–F)** red peaks are b-ions, blue peaks are y-ions and violet vertical lines mark parent/precursor masses and b/y ladder ends.

Comparison of the mass-spectrum of the synthetic peptide KLVVGAVGV (that represents the presumed spliced peptide) to spectra identified by the authors as KLVVGAVGV in 34 out of the 45 runs indicates that the spectra are sufficiently different and that the identifications of these spectra in these runs were possibly incorrect. **Figure 1A** below shows a spectrum of synthetic KLVVGAVGV derived from the authors’ data. **Figures 1B–D** show three spectra that faithfully represent all the spectra identified by the authors as KLVVGAVGV in the 34 runs (identification results are in files F011920.csv, F011921.csv and F011929.csv in PRIDE project PXD015580). The three spectra are of scans 11558, 11683 and 11389 in MS run j_liepe_db5_2_qex1_100519_rep3 (in PXD015580) derived from biological replicate 3, technical replicate 3, time-point 4 hours. Interestingly, these spectra share the same m/z and have similar retention times but belong to three different HPLC elution envelopes in the same MS run (data not shown). None of the three spectra are identical to the spectrum of the synthetic peptide in **Figure 1A**. In addition, the spectra in **Figures 1B, C** belong to peptides that already exist in the samples of 0 hours (e.g., in run j_liepe_db1_2_qex1_100519_rep3 scans 11415 and 11561 respectively) and hence are not digestion products. The spectrum in **Figure 1D** possibly belongs to a digestion product as will be discussed below.

## A Possible Alternative Identification Without Relying on the Peptide Splicing Hypothesis

We suggest that the spectrum in **Figure 1D** represents the **non-spliced** KRAS G12V peptide KLVVVGAVG. This peptide has the same mass as the presumed spliced peptide KLVVGAVGV and was among the possible identifications of the spectra in question by the Mascot search engine (e.g. in file F011929.csv). However, the authors opted not to choose this identification. **Figure 1E** shows the same spectrum of **Figure 1D** with the alternative assignment to the non-spliced peptide KLVVVGAVG. To confirm this alternative identification, we synthesized KLVVVGAVG and ran MS analysis of this peptide. **Figure 1F** shows the spectrum of the synthetic KLVVVGAVG, which is similar to the spectrum in **Figure 1E**, supporting our suggested alternative identification.

## The Spliced Peptide Was Found in Follow Up Analyses by the Authors but Might Have Resulted From Contamination

Based on the results of the 45 MS runs, and on the assumption that the “spliced” KLVVGAVGV was identified correctly, the authors performed further analysis of this peptide, including obtaining its synthetic version for quantitation. In parallel to the quantitation experiments, they also analyzed by MS two samples that resulted from 20 hours of digestion by proteasomes. This took place several weeks after the analysis of the original 45 samples.

Despite not being found (according to our analysis) in any of the initial 45 MS runs, the spliced peptide KLVVGAVGV was found and identified correctly in the two runs of 20-hours digest. In these runs, the obtained spectra appeared identical to the synthetic version (data not shown). A possible explanation could be longer digest and accumulation time. However, according to Figure 4D of the paper being discussed, digestion reached saturation after 3-4 hours. Our possible alternative explanation for finding the spliced peptide in these late runs could be contamination of the 20-hours samples by the synthetic peptide. At the time when the 20-hours samples were run, the authors already obtained the synthetic peptides and performed titration measurements with up to 10 pmol (a very large amount for a single peptide). This could have resulted in carry-over in the HPLC/MS or in sample cross-contamination during preparation.

To conclude, our analysis may suggest that the *in vitro* proteasome digestion products did not include the spliced peptide and therefore further analysis relied on incorrect identification. In our opinion, the obtained spectra could be explained without relying on hypothesized splicing in the proteasome.

## Author Contributions

IB performed the analysis and wrote this commentary. All authors contributed to the article and approved the submitted version.

## Conflict of Interest

Author IB was employed by the company Adicet Bio Inc.
